# Consumer Willingness to Pay for Hybrid Food: The Role of Food Neophobia and Information Framing

**DOI:** 10.3390/nu17142326

**Published:** 2025-07-16

**Authors:** Siwei Chen, Dan Wang, Jingbin Wang, Jian Li

**Affiliations:** 1 College of Economics and Management, Huazhong Agricultural University, Wuhan 430070, China; csw45@webmail.hzau.edu.cn (S.C.); wangjingb@webmail.hzau.edu.cn (J.W.); jli@mail.hzau.edu.cn (J.L.); 2 Research Center of Food Economics, Nutrition and Health, Huazhong Agricultural University, Wuhan 430070, China

**Keywords:** hybrid foods, willingness to pay, food neophobia, information framing

## Abstract

**Background/Objectives:** The global food system faces mounting pressures from population growth, dietary transitions, and resource and environmental constraints. Hybrid foods, which combine nutritional, environmental, and economic advantages, are increasingly regarded as a promising solution. This study examined consumer acceptance and willingness to pay (WTP) for a novel hybrid food product—beef rice. **Methods:** Based on online survey data collected from 1536 Chinese consumers, this study measured food neophobia and investigated its influence on WTP for beef rice. In addition, it explored the moderating effects of four distinct types of information interventions. **Results:** More than 80% of respondents expressed a willingness to purchase beef rice. Food neophobia exerted a significant negative effect on WTP (β = –1.538, *p* < 0.001). Among the information treatments, environmental information significantly mitigated the negative impact of food neophobia on WTP (β = 0.573, *p* < 0.01), while health-related and combined framings did not show significant effects. **Conclusions:** Chinese consumers generally hold a positive attitude toward hybrid foods such as beef rice. However, food neophobia significantly reduces their WTP. Environmental information shows a significant moderating effect and may serve as an effective strategy to enhance consumer acceptance of novel hybrid food products.

## 1. Introduction

The global food system is confronted with numerous challenges, such as population growth, dietary transition, and constraints related to resources and the environment. According to the United Nations [1], the global population is projected to surpass 9.7 billion by 2050, with food demand anticipated to rise by approximately 70%. Among these drivers, the rapid growth in demand for animal protein emerges as a pivotal factor. For instance, in China, total meat production reached 97.48 million tons in 2023, reflecting a year-on-year increase of 4.5%. Specifically, beef production reached 7.52 million tons, indicating a 4.8% increase [2]. 

In the context of supply–demand mismatches, the resource consumption and environmental externalities associated with traditional livestock farming have become increasingly significant. Studies indicate that beef production accounts for up to 41% of total agricultural carbon emissions [3], while global beef consumption availability is projected to increase by approximately 5.9% by 2030 [4]. Given the limited environmental capacity, the increasing production of protein intensifies the sustainability challenges within agricultural systems. This has prompted both academic and industrial sectors to actively investigate novel protein alternatives. How to achieve a dynamic balance between ensuring nutritional safety and protecting ecosystems via technological innovation has become a critical issue requiring immediate attention in the field of global food governance.

Hybrid foods are made by blending animal-based and plant-based ingredients to make food products that are similar to the 100% animal-based ones [5]. “Beef rice”, an emerging hybrid food, exemplifies novel protein integration technology and has garnered growing attention. This product enables the directional growth of bovine muscle stem cells and facilitates functional integration with the rice matrix by constructing a three-dimensional scaffold on the surface of rice grains. Experimental results indicate that the protein content of beef rice is 8.66% higher than that of ordinary rice, while the carbon emission per 100 g of protein is significantly lower than that of traditional beef (49.89 kg) and even lower than rice (6.27 kg) [6]. Beef rice realizes the technological integration of health enhancement and carbon emission reduction, showcasing substantial functional and environmental substitution potential, and constitutes a valuable exploration for constructing a sustainable food system. Although the technical feasibility and environmental benefits have been preliminarily validated, quantitative analysis regarding consumer acceptance, particularly WTP, remains inadequate, thereby impeding the development of marketization strategies and policy promotion for these products.

Despite these benefits, consumer acceptance remains a key barrier. The commercialization of beef rice may be hindered by consumer psychological barriers, including food neophobia [7]. This refers to individuals’ negative expectations and rejection attitudes toward unfamiliar foods [8]. Such attitudes may negatively affect hybrid foods, like beef rice, which possess prominent technical and perceived artificiality. In the field of food neophobia research, existing studies have encompassed a variety of food categories [8]. The research scope encompasses a variety of countries, including the United States [9], Ireland [10], Sweden [11], and Finland [12].

The existing literature primarily focused on the technical aspects of arguing for the potential of hybrid foods. Few studies systematically investigated the market acceptance of these products from the perspectives of consumer preferences and behavioral psychology. Particularly, there are certain deficiencies in the empirical modeling of payment willingness. For example, hybrid foods such as beef rice that possess dual functional values are rarely subject to research that quantifies their economic value at the micro-level of consumer choice, thereby creating an information gap for the market pricing and policy support of related products. In addition, while the existing literature has identified the negative impact of food neophobia on the acceptance of everyday foods, there remains a lack of research specifically addressing particular cultural contexts (e.g., China) and hybrid foods (e.g., beef rice). Moreover, positive attribute framing can often alleviate consumers’ fear of unfamiliar foods and may regulate the negative impact of food neophobia on hybrid foods such as beef rice.

Based on this, this study contributes in two aspects: first, by leveraging indicators such as consumers’ WTP, this paper quantitatively assessed Chinese consumers’ acceptance of hybrid foods, using the Chinese market and beef rice as case examples. This not only fills gaps in the identification of micro-preferences and market response mechanisms for such foods but also provides an empirical basis for designing targeted commercialization strategies and informing future government interventions. Second, this study investigated the impact of food neophobia on consumers’ WTP for hybrid foods like beef rice, and further introduces a health and environmental information framework to examine its moderating effect on consumer cognition and food choice behavior.

## 2. Literature Review and Hypotheses

### 2.1. Consumer WTP for Hybrid Food: Measurement and Evidence

Hybrid foods primarily consist of hybrid rice (e.g., beef rice), hybrid meat (e.g., sausages, meatballs), and 3D-printed food (e.g., 3D-printed steak). The existing literature on hybrid foods remains in the early stages of fragmented investigation, and no consensus has been reached regarding consumer acceptance of such products. Research on consumer attitudes toward hybrid meats and their WTP also remains limited and underdeveloped [13]. Consumers may perceive hybrid foods as ambiguous or uncertain, particularly when sensory experiences fail to align with their expectations. This perceived discrepancy can lead to reduced willingness to pay a premium for such products [5,14]. Indeed, consumers are more likely to pay a premium only when they recognize the quality advantages, such as texture, of 3D-printed food [15]. The beef rice examined in this study is a hybrid food that integrates animal protein with plant-based grains, offering high nutritional density and potential environmental benefits. Theoretically, it can be categorized as “hybrid food”, yet no systematic quantitative research has been conducted on its willingness-to-pay aspect. Building on these findings, we hypothesize the following:

**H1.**  *Consumers exhibit a positive WTP for beef rice.*

To quantify consumers’ WTP for hybrid foods, the academic community frequently employs two primary paradigms: the first is the stated preference method, and the mainstream methods for measuring it include the contingent valuation method (CVM) and the discrete choice experiment (DCE). The CVM has been utilized in simulated market scenarios to estimate WTP within the agribusiness sector [16]. Additionally, consumers’ WTP was significantly lower in open-ended contingent valuation formats compared to discrete choice experiments [17]. The second methodology is incentive-compatible mechanisms, such as the Becker–DeGroot–Marschak (BDM) method, which assesses consumers’ intrinsic valuation of goods in real transaction environments [18,19]. This study employed the stated preference method and the payment card approach. The primary rationale is to examine the impact of food neophobia on consumers’ WTP. Additionally, in contrast to the multi-attribute interaction analysis of DCE, CVM simplifies attribute weights by using direct valuation questions, thereby reducing the cognitive burden on respondents when evaluating novel foods. Lastly, the payment card method establishes a predefined price range, offering a valuation anchor for hybrid foods that lack market references while ensuring a balance between data accuracy and distribution controllability.

### 2.2. Food Neophobia

The term “neophobia” was first introduced into the literature in 1958 [20], formally defining the “new object reaction” as an expression of neophobic behavior in previously study [21]. Furthermore, neophobia can also be characterized by a reduction in an animal’s food intake when familiar food is replaced with novel food, which may be attributed to the avoidance of unfamiliar stimuli [20]. Neophobia was initially primarily applied in the study of animal psychology and behavior [22,23]. Subsequently, research on food neophobia gradually expanded and became a central component of this field.

Food neophobia has since been extensively employed to elucidate the variations in consumers’ acceptance of functional foods and traditional foods [24,25]. Its research scope encompasses a variety of specific populations, including children, school-aged girls, and college students [9,11,26]. The food neophobia scale (FNS) was developed to quantify individuals’ resistance to new foods through 10 assessment items [7]. Subsequent studies have predominantly utilized Likert scales to quantitatively assess consumers’ levels of food neophobia [8,27]. A higher score on the FNS indicates lower consumer acceptance of unfamiliar foods [28], and such consumers may only be willing to purchase unfamiliar foods when these are discounted or on sale. As a result, consumers with higher food neophobia scores tend to avoid novel foods, especially when higher prices are involved. Therefore, this paper proposes the following hypothesis:

**H2.**  *Consumers’ food neophobia tendency is negatively correlated with their WTP for beef rice.*

### 2.3. The Moderating Role of Information Framing

The moderating effect of information framing on consumers’ WTP may stem from its capacity to restructure cognitive processes and shape individuals’ assessment of potential benefits in relation to opportunity costs. Drawing on Regulatory Focus Theory [29], the processing context of positive informational framing can mitigate the negative impact of risk on overseas direct purchasing intentions [30]. Consequently, we propose that information framing influences consumers’ WTP by selectively activating the prevention or promotion motivational systems, thereby adjusting their psychological boundaries toward novel products.

Similarly, hybrid food products such as beef rice currently fall under a regulatory gray area. In jurisdictions such as the European Union and China, such products would likely require approval under novel food regulations due to their compositional and technological characteristics. Against this backdrop, the manner in which information is framed can influence consumers’ perceptions of such products. More specifically, a prevention-oriented frame (e.g., highlighting safety inspections and guarantees) may mitigate safety concerns; however, if overemphasized, it could inadvertently elevate the threshold for payment decisions. In contrast, a promotion-oriented frame (e.g., emphasizing health benefits and health certifications) may engage the promotion motivational system within Regulatory Focus Theory by shifting consumer attention to potential gains, thereby effectively counteracting the inhibitory effects of neophobia. Based on these insights, we formulate the following moderating hypotheses:

**H3a.**  *Health-related information mitigates the adverse effect of neophobia on WTP.*

**H3b.**  *Environmentally friendly information attenuates the negative influence of neophobia on WTP.*

**H3c.**  *The integrated framework of health and environmental information exhibits a stronger regulatory effect on neophobia.*

## 3. Research Methods

### 3.1. Experimental Design

This study utilized survey data from Chinese online consumers and adopted a systematic experimental design to ensure the validity of the data. The questionnaire was developed through focus group interviews and three rounds of pretesting, with refinements made based on feedback. Finally, by employing a stratified random sampling method across the country based on the population proportions of each province from the Seventh Chinese National Population Census [31], the sample was ensured to be representative in terms of regional and demographic characteristics. The formal survey was scheduled to take place from September to November 2024. We designed the survey using the Qualtrics platform and administered it through “Le Diaocha”, a leading online survey service provider in China, which has supported multiple consumer behavior research projects [32]. The respondents were primary decision-makers for household food purchases, thereby ensuring the representativeness and relevance of the data.

The questionnaire design encompassed four core modules: First, by analyzing consumers’ traditional rice purchasing behaviors (including purchase prices, etc.), their basic consumption habits were assessed. Second, an “introduction and information intervention for beef rice” module used randomized experimental groups to evaluate the impact of different information treatments on WTP (see Section 3.2.3 for details). Third, through the “purchase intention and WTP for beef rice” module, consumer behavioral tendencies toward the product were assessed using the payment card method to quantify WTP (see Section 4.2 for a detailed analysis). Finally, basic demographic information of the sample (e.g., age, income, and educational level) was collected to control for potential confounding variables (see Section 4.1 for a detailed analysis). To ensure data quality, the questionnaire design included trap questions aimed at detecting and excluding invalid responses, resulting in a final total of 1536 valid questionnaires.

### 3.2. Measurement of Variables

#### 3.2.1. Dependent Variable

The dependent variable in this study is consumers’ WTP for beef rice. Participants were informed that the average price of traditional rice in the current Chinese market is approximately 3.5 yuan per jin (500 g), which served as a reference point. The WTP for beef rice was assessed using the following scenario-based question:

“Suppose there is a 5-jin package of traditional rice available in stores at 3.5 yuan per jin (500 g). Now, consider a 5-jin package of ‘beef rice’ (containing 2.4 g of beef cells per jin). Would you be willing to purchase it?

A. Yes, I am willing to purchase ‘beef rice’.
B. No, I am not willing to purchase ‘beef rice’.”

If participants selected “Yes”, their WTP was further evaluated using the Payment Card Method. This method required participants to choose an appropriate payment range from a set of predefined price intervals. The payment card consisted of 19 price intervals, ranging from 0 to 9 yuan, with each interval covering a span of 0.5 yuan. If a participant’s WTP exceeded 9 yuan, they could select the “9 yuan or higher” option. Additionally, participants who were unwilling to make a purchase could choose the “not purchase” option. An example of beef rice can be found in a previous study [6].

#### 3.2.2. Independent Variables

The core independent variable in this study is consumers’ fear of novel foods, which was measured using the FNS. The scale comprises 10 statements (see the table in Section 4.3.2), which is designed to assess consumers’ fear of unfamiliar foods. Based on focus group interviews and pre-research feedback, certain vocabulary and sentence structures were adaptively modified to better align with the cultural and living environment of Chinese consumers. The scale uses a five-point Likert scale (1 = strongly disagree, 5 = strongly agree) for scoring. The score for each statement reflects the consumer’s attitude toward novel foods. A lower total score indicates greater acceptance of novel foods. The overall food neophobia score was calculated by summing the scores across all items.

#### 3.2.3. Moderating Variable

To examine the moderating effect of information framing type on WTP, this study established four randomized control groups with distinct framing conditions. We provided basic information regarding beef rice to consumers in four distinct consumer groups. Subsequently, the second, third, and fourth groups were provided with additional information frames focusing on health benefits, environmental benefits, and a combination of both health and environmental benefits, respectively. All information content strictly follows the framework outlined in the previous literature [6]. A detailed description of the information content is shown in Table 1.

#### 3.2.4. Control Variables

Demographic variables such as gender, residence, age, family size, education level, and annual household income may influence WTP for food. To ensure a comprehensive analysis, gender, residence, age, and family size were included as control variables. Education and pre-tax household income were represented by sets of dummy variables to assess their effects. Since different information interventions may result in variations in consumers’ WTP, this study established three dummy variables for health information processing, environmental information processing, and combined health and environmental information processing. The moderating effects of these variables on WTP were subsequently examined through empirical analysis. The specific meanings and values of all variables are presented in Table 2.

### 3.3. Econometric Foundations

#### 3.3.1. Econometric Models

(i) Interval Censored Regression Model

In the payment card-based contingent valuation, WTP is interval-censored, reflecting an underlying continuous variable observed in discrete intervals. The application of Logit or Probit models necessitates the conversion of WTP into binary or ordered outcomes, thereby introducing specification errors due to the disregard of its continuous nature. These models estimate the probability of choosing a given interval but cannot directly assess the marginal effects of covariates on latent WTP or utilize within-interval variation, reducing statistical efficiency. Therefore, we employed an interval-censored regression model to analyze respondents’ selected price intervals. Following the previous approach [27], we first developed a simple regression model:

(1) $${WTP}_{i}=\beta X_{i}+\epsilon_{i}$$

here, ${WTP}_{i}$ represents the potential actual willingness-to-pay price of the respondent, which is unknown but must fall within the range of the willingness-to-pay price they have chosen; $X_{i}$ is a vector of individual-specific explanatory variables; *β* is a consistent vector of parameters to be estimated; and $\epsilon_{i}$ is an error term that is independent and identically distributed across individuals.

Define a set of known price-range boundaries $\alpha_{1}{<\alpha }_{2}{<\alpha }_{3}{<\cdots <\alpha }_{k}$, which divide the prices into different intervals. The probability that respondent *i* selects the interval [$\alpha_{j-1},\;\;\alpha_{j}$] is as follows:

(2) $${Pr}_{i}(\alpha_{j-1}{<WTP}_{i}{<\alpha }_{j})={Pr}_{i}({WTP}_{i}<\alpha_{j})-{Pr}_{i}({WTP}_{i}<\alpha_{j-1})$$

The above equation is transformed into the calculation of the cumulative distribution function of the standardized normal distribution = $\Phi (\frac{\alpha_{j}-\beta X_{i}}{\sigma })-\Phi (\frac{\alpha_{j-1}-\beta X_{i}}{\sigma })$. If respondent *i* selects the highest price range of “9 yuan and above”, the probability of this choice is expressed as follows:

(3) $$\;\;{Pr}_{i}(\alpha_{k}{<WTP}_{i})=1-\Phi (\frac{\alpha_{k}-\beta X_{i}}{\sigma })$$

Furthermore, the probability that respondent *i* selects “unwilling to purchase” is ${Pr}_{i}({WTP}_{i}<0)=\Phi (\frac{-\beta X_{i}}{\sigma })$.

The likelihood function *L* used to estimate the coefficient *β* is the product of the selection probabilities of all respondents:

(4) $$L=\prod_{i=1}^{n}\prod_{j=1}^{k}\left[\Phi (\frac{\alpha_{j}-\beta X_{i}}{\sigma })-\Phi (\frac{\alpha_{j-1}-\beta X_{i}}{\sigma }\right){]}^{I({WTP}_{i}=j)}$$

In the formula, $I({WTP}_{i}=j)$ is an indicator function, which equals 1 when the respondent selects the jth price range, and 0 otherwise.

All the model estimations and predictions mentioned above were implemented in Stata 17 using the intreg command.

(ii) Moderating Effect Test Model

This study employed an interval-censored regression model to address the truncated data characteristics of WTP. The dependent variable is the acceptable price range (minimum to maximum value) that consumers choose for beef rice. The model can be expressed as follows:

(5) $$WTP=\beta_{0}+\beta_{1}FNS+\beta_{2}X+\varepsilon$$

In Equation (5), *WTP* represents the consumers’ WTP for beef rice, *FNS* represents the consumers’ scores on the food neophobia scale, *X* represents the other control variables, and *ε* indicates the error term. Among them, the set of control variables includes demographic variables such as age, income, and education level.

For testing the moderating effect on information processing, a regression model is established:

(6) $$WTP=\beta_{0}+\beta_{1}FNS+\beta_{2}X+\beta_{3}infor+\beta_{4}FNS\times infor+\varepsilon$$

where WTP represents the consumers’ WTP for beef rice, FNS represents the consumers’ scores on the food neophobia scale; X represents other control variables; infor represents the three types of information processing; FNS×infor represents the moderating effect of information processing, reflecting the moderating intensity of the information frame on the neophobia effect; and ε indicates the error term. In statistical inference, simple slope analysis is used to quantify the differences in the marginal effects of neophobia on WTP under different information frames.

#### 3.3.2. Robustness Test Model

To verify the reliability of the model estimation, a multivariate Tobit model was employed to replace the original model for robustness testing. The median value of the consumers’ willingness-to-pay interval was used as the dependent variable to construct the Tobit model, which handles both left and right censoring. The specific model construction followed established methodologies outlined in prior research [33]. The generation rule for the two left-censored dependent variables at zero and the observed variables in the multivariate Tobit model can be expressed as follows:

$$y_{i}^{*}=X_{i}\beta +\varepsilon_{i},\;\varepsilon_{i}\sim N(0,\;\sigma^{2})$$

(7) $$\;\;y_{i}=\left\{\begin{array}{c} y_{i}^{*}\;\;\;if{\;\;\;y}_{i}^{*}>0\\ 0\;\;\;\;\;if{\;\;\;y}_{i}^{*}\leq 0 \end{array}\right.$$

where $y_{i}^{*}$ is a latent variable that is only observed when positive, and $y_{i}$ represents the consumer’s WTP for beef rice; $X_{i}=[1,X_{i1},X_{i2}]$ is the vector of independent variables; β is the vector of parameters to be estimated; $\varepsilon_{i}$ is a normally distributed error term with an independent and identical distribution. The Tobit model solves for parameters using MLE as shown in Equation (8), with the algorithm in Equation (9).

(8) $$L\left(\beta ,\sigma \right)=\prod_{y_{i}>c}\frac{1}{\sigma }\emptyset (\frac{y_{i}-X_{i}\beta }{\sigma })\times \prod_{y_{i}=c}\Phi (\frac{c-X_{i}\beta }{\sigma })$$

(9) $$\ln L\left(\beta ,\sigma \right)=\sum_{yi>c}\left[-\frac{1}{\sigma }\emptyset \left(\frac{y_{i}-X_{i}\beta }{\sigma }\right)\right]+\sum_{yi>c}\ln\Phi \left(\frac{c-X_{i}\beta }{\sigma }\right)$$

## 4. Analysis of WTP for Beef Rice and Consumers’ Food Neophobia

### 4.1. Analysis of Sample Characteristics

This study obtained 1536 valid responses via stratified random sampling. Sample characteristics are reported in Table 3. Respondents were 61.2% male and 38.8% female, broadly consistent with national census data [2]. Most held a bachelor’s degree or higher (92%), were aged 30–60 (70%), and had annual household incomes between 200,000 and 400,000 CNY (43%). Given their education and purchasing power, middle-aged, well-educated individuals are more likely to be potential consumers of hybrid foods like beef rice. In addition, the demographic characteristics of our sample are broadly consistent with those reported in other online food consumer surveys conducted in China [34].

### 4.2. Analysis of WTP for Beef Rice

**Figure 1:**
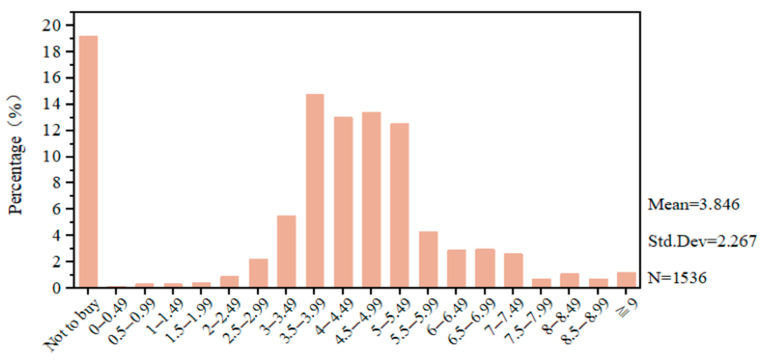
Overall distribution of consumers’ WTP for beef rice. The horizontal axis represents consumers’ WTP for beef rice, expressed in Chinese CNY (CNY) per 500 g, while the vertical axis represents the percentage of respondents selecting each corresponding payment interval relative to the total sample.

Figure 1 presents the overall descriptive statistics of consumers’ WTP based on responses from 1536 individuals. To approximate each respondent’s actual WTP, the midpoint of each price interval was used. The average WTP was 3.846 CNY per jin (500 g), reflecting a 16.4% premium over the 2024 national average market price of traditional rice (3.303 CNY per jin) [35]. More than 80% of consumers reported a willingness to purchase beef rice, suggesting strong market acceptance. Consequently, we fail to reject H1. In terms of frequency distribution, aside from the 19.2% of respondents who indicated no willingness to purchase, the most commonly selected WTP range was 3.5–3.99 CNY per jin (14.7%), followed by 4.5–4.99 CNY (13.4%), 4.0–4.49 CNY (13.0%), and 5.0–5.49 CNY (12.5%). These results suggest the presence of a potential market premium for beef rice. These findings are consistent with prior studies showing consumer WTP at a premium for hybrid food products. For example, Indian university students were willing to pay 46.7 INR/kg for Golden Rice, a 4% premium over the conventional price of 45 INR/kg [36]. The higher WTP observed in our study may be attributed to the sample’s demographic profile, which consists largely of middle-aged consumers with higher purchasing power than students.

### 4.3. Analysis of Consumers’ Food Neophobia

#### 4.3.1. FNS Scores

**Figure 2:**
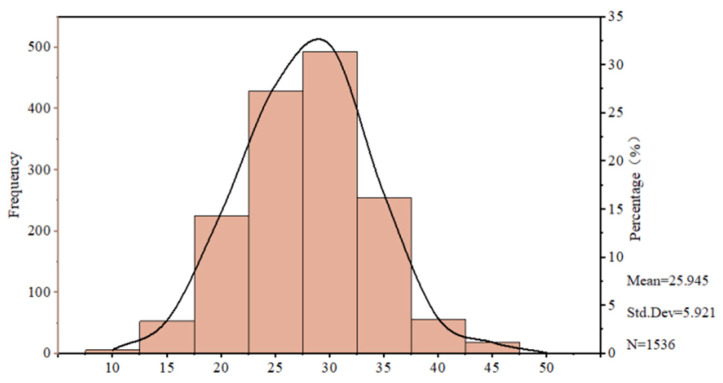
Distribution of overall scores on the FNS scale. The horizontal axis shows consumers’ scores on the FNS. The primary vertical axis displays the number of consumers at each score as bars. The secondary vertical axis shows the proportion of individuals in each score range relative to the total sample, indicated by a trend line.

Figure 2 presents the distribution of FNS scores derived from methodologies established in prior research [37]. Items 1, 4, 6, 9, and 10 were reverse-coded; total scores range from 10 to 50. The distribution appears left-skewed, with a mean of 25.95 (SD = 5.92), and most scores fall below the neutral threshold of 30, indicating generally positive attitudes toward novel foods. Some studies have demonstrated that consumers from different countries exhibit varying FNS scale scores and differing levels of fear of new foods. When using a 7-point Likert scale to measure the FNS score, Spanish consumers had an average FNS score of 31.74 (SD = 10.98) [38]. Korean respondents scored an average of 36.4 (SD = 11.86) [28], while American students had a slightly lower average FNS score of 29.8 (SD = 11.7) [39]. Consequently, we conclude that consumers’ cultural background likely plays a role in influencing their level of food neophobia.

**Figure 3:**
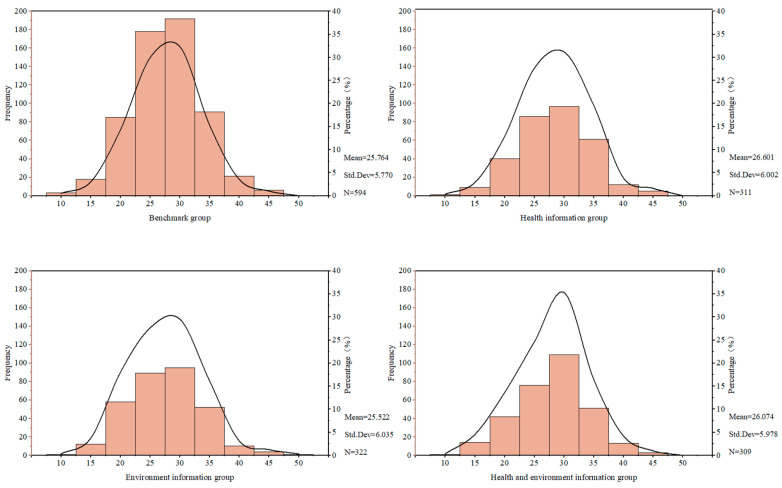
Distribution of FNS scores across groups. The horizontal axis shows consumers’ scores on the FNS. The primary vertical axis displays the number of consumers at each score as bars. The secondary vertical axis shows the proportion of individuals in each score range relative to the total sample, indicated by a trend line.

Figure 3 presents the FNS scale scores of consumers grouped by statistical analysis. Overall, the bar charts of the four data groups show a relatively high frequency in the middle score range (25–30 points), suggesting that each group of data demonstrates a certain degree of concentration and that consumer attitudes within each group tend to be positive. By comparing the FNS scores across the different information processing groups, the health information processing group achieved the highest score, with an average of 26.60, reflecting the highest level of fear of new things. This was followed by the health environment information group (26.07 points), the benchmark information group (25.76 points), and the environment information group (25.52 points). The distribution range of FNS scale scores and the proportion of consumers within each range for the four groups are consistent with the overall pattern.

#### 4.3.2. Two-Factor Partitioning of the FNS Scale

To more comprehensively analyze the impact of FNS on consumers’ WTP, this paper performs a factor analysis on the scores of the ten items, aiming to examine the influence extent and direction of several factors from multiple dimensions. For specific details, please refer to Table 4. Principal component analysis with varimax rotation was conducted, extracting factors with eigenvalues greater than one. Based on the results of the exploratory factor analysis (EFA), it is recommended to divide the structure into two factors. Among these, items 1, 4, 6, 9, and 10 are classified under food neophilia (indicating a greater willingness to try new foods), while items 2, 3, 5, 7, and 8 are categorized under food neophobia (suggesting a higher level of fear towards new foods). The means and standard deviations of respondents’ scores on each item, as well as the results of the exploratory factor analysis, are presented in Table 4.

**Figure 4:**
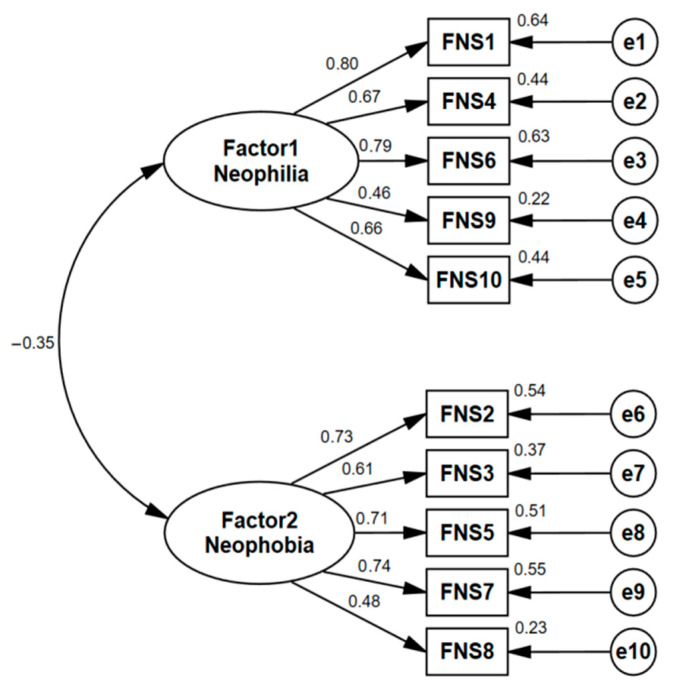
The best-fitting model of the two factors of FNS. Numbers within rectangles represent FNS item IDs and measured variables. Ellipses represent latent constructs. Numbers above vectors indicate item-construct correlations; those above rectangles indicate the predictive power (R^2^) of each item.

Figure 4 displays the division results of the two factors. Specifically, the two factors are negatively correlated (r = −0.35), which statistically suggests that consumers’ willingness to try new foods and their avoidance tendencies exhibit an asymmetric complementary relationship. In other words, as an individual’s willingness to try new foods increases, their avoidance tendency toward unfamiliar foods may decrease. Most prior studies have identified that the FNS is loaded on two factors, although the specific items within each factor may vary. The classification results in this study align with previous findings [27,40,41], with the exception that item 5 or item 9 may differ in terms of whether they are included in the factor analysis results.

(i) Item Testing

To further refine the items, the pretest data were evaluated using corrected item-total correlations and Cronbach’s alpha. All items exceeded the thresholds (CITC ≥ 0.4; Cronbach’s alpha ≥ 0.7), indicating acceptable reliability and internal consistency.

(ii) Factor Analysis

We conducted exploratory and confirmatory factor analyses to evaluate the construct validity of the scale. KMO values (0.836 and 0.817) and a significant Bartlet’s test confirmed the data’s suitability for factor analysis. Principal component analysis with varimax rotation extracted two factors with eigenvalues > 1, explaining 56.77% of the total variance (Table 5). Factor loadings (Table 6) were satisfactory. Confirmatory factor analysis using AMOS and maximum likelihood estimation showed good model fit (GFI = 0.961, CFI = 0.948, NFI = 0.941, TLI = 0.931, IFI = 0.948; all > 0.90; RMSEA = 0.07 < 0.08), supporting the proposed measurement model.

(iii) Reliability and Validity Test

This study further examined the convergent validity and composite reliability of the model. The Cronbach’s α coefficients, as shown in Table 5, ranged from 0.7 to 0.9. The composite reliability (CR) values, displayed in Table 6, were all above 0.7, and the average variance extracted (AVE) values were close to 0.5. These results confirmed that the FNS exhibited good composite reliability and convergent validity. Therefore, this study confirmed the two-factor original model as the final structure and item configuration of the consumer food neophobia scale.

#### 4.3.3. Analysis of the Two-Factor Score Situation

**Figure 5:**
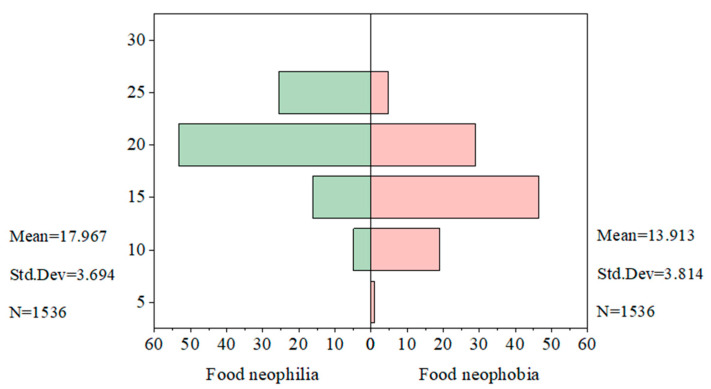
Descriptive statistics of two-factor scores. The horizontal axis shows consumers’ scores on the sub-dimensions of the FNS, while the vertical axis indicates the proportion of consumers corresponding to each score.

Figure 5 presents the frequency distributions of the two-factor scores in the FNS scale. The horizontal axis represents the percentage distribution of the number of consumers within each score range, while the vertical axis indicates the score situation of the FNS scale for consumers. Each factor ranges from 5 to 25 points. The food neophilia scores are primarily concentrated above the midpoint (15), indicating a generally positive attitude and strong willingness to try new foods. In contrast, the food neophobia scores are mostly below 15, suggesting low levels of avoidance toward unfamiliar foods. Overall, consumers exhibit high openness to beef rice.

## 5. Empirical Results and Discussion

This study first employed an interval-censored model for benchmark regression to estimate the impact of food neophobia and its two factors on consumers’ WTP for beef rice. The results are presented in Table 7. A mean analysis of the core explanatory variables was conducted, and interaction terms of the three types of information interventions were further incorporated to test the moderating effect, with the results displayed in Table 8. Finally, robustness tests were performed using model and sample replacements, and the results are summarized in Table 9 and Table 10.

### 5.1. Benchmark Regression Analysis

Columns (1) and (2) of Table 7, respectively, present the regression results based on the summed scores of the FNS scale and those obtained after dividing it into two factors. Both models show good fit (Log Likelihood = −4124.388 and −4124.251; Wald χ^2^ = 206.15 and 207.80; *p* < 0.01). The FNS coefficient is significantly negative at the 1% level, indicating that food neophobia significantly reduces consumers’ WTP for beef rice. H2 is not rejected.

Specifically, the higher the degree of food neophobia among consumers, the lower their WTP for beef rice. Notably, food neophilia exhibits a significantly positive relationship with WTP, indicating that the willingness to try new foods is a significant positive driver of payment intentions. In contrast, food neophobia shows a significantly negative relationship, implying that stronger resistance or fear toward unfamiliar foods reduces consumers’ WTP. Together, these dual effects jointly shape consumers’ willingness-to-pay levels. This regression result demonstrates that consumer FNS significantly suppresses their WTP for beef rice. The underlying mechanism involves the positive driving force of food neophilia and the negative inhibitory effect of food neophobia, which together reveal the dual-dimensional motivations underlying consumers’ payment decisions.

**Table 7:** Benchmark regression results.

(1)			(2)		
	Coef.	Robust Std.Err.		Coef.	Robust Std.Err.
FNS	−1.538 ***	(0.120)	Neophilia	0.730 ***	(0.098)
			Neophobia	−0.806 ***	(0.091)
Income 2	0.347 **	(0.156)	Income2	0.351 **	(0.156)
Income 3	0.756 ***	(0.246)	Income3	0.760 ***	(0.246)
Income 4	1.098 ***	(0.223)	Income4	1.087 ***	(0.226)
College	0.856 ***	(0.329)	College	0.856 ***	(0.328)
Graduate	0.569	(0.410)	Graduate	0.572	(0.409)
Age 2	0.013	(0.150)	Age2	0.018	(0.151)
Age 3	0.875	(0.682)	Age3	0.860	(0.681)
Gender	−0.072	(0.145)	Gender	−0.071	(0.145)
Log Likelihood	−4124.388		Log Likelihood	−4124.251	
Wald (*p*-value)	206.15 (0.00)		Wald (*p*-value)	207.80 (0.00)	
N	1536		N	1536	

Notes, *** and ** indicate significance at the 1% and 5% levels, respectively; The values in parentheses are robust standard errors.

As shown in the first two columns of Table 7, Income 2–Income 4 are positively associated with WTP, with Income 3, Income 4, and College significant at the 1% level and Income 2 at the 5% level. These results indicate that income and household size have a significant positive impact on WTP. This may be attributed to their heightened attention to food safety and nutritional attributes, which drives their payment decisions beyond the general level, forming a “quality–price” trade-off preference [42]. Furthermore, consumers with a college degree are also more willing to pay, possibly due to higher purchasing power and better information access. Notably, the coefficients of gender and age on WTP did not reach the threshold for statistical significance, reflecting the modernized convergence characteristics of food consumption behavior.

### 5.2. Moderation Effect Test

The first two columns of Table 8 present the results of the moderation effect tests for the FNS scale after score summation and when it is divided into two factors. In the models containing interaction terms, to avoid multicollinearity between the independent variables, moderating variables, and their interaction terms, the data of the explanatory variables were mean-centered. The models show good fit, with Log Likelihoods of −4118.94 and −4114.02 and Wald statistics of 218.89 (*p* < 0.001) and 224.66 (*p* < 0.001), respectively. As shown in column (1), the interaction between FNS and environmental information is significantly positive, indicating that environmental cues attenuate the negative effect of food neophobia on WTP, thereby H3b cannot be rejected. In contrast, the interactions involving health information and combined health and environmental information do not reach statistical significance. Consequently, we reject H3a and H3c.

As shown in Column (2), the interaction between food neophilia and information processing is not significant. However, the interaction terms between food neophobia and both health and environmental information are significantly positive, suggesting that these information types mitigate neophobia and thereby increase WTP. This finding contrasts with previous studies that identified neophilia as the dominant factor in novel food acceptance, with neophobia exerting only minor effects [43]. One possible explanation is that external stimuli such as health and environmental messages reduce aversion to unfamiliar foods rather than directly enhancing novelty-seeking tendencies, thereby amplifying the influence of neophobia. Significance patterns for demographic controls remain consistent with the baseline results, despite minor coefficient shifts.

**Table 8:** Results of moderating effect test.

	(1)			(2)	
	Coef.	Robust Std.Err.		Coef.	Robust Std.Err.
FNS	−1.869 ***	(0.196)	Neophilia	0.760 ***	(0.113)
			Neophobia	−0.824 ***	(0.119)
Health	0.444 **	(0.183)	Health	0.454 **	(0.182)
Envir	0.297	(0.188)	Envir	0.293	(0.188)
Health + Envir	0.232	(0.189)	Health + Envir	0.229	(0.189)
FNS × Health	0.531	(0.331)	Neophilia × Health	−0.122	(0.187)
FNS × Envir	0.573 *	(0.321)	Neophobia × Health	0.323 *	(0.189)
FNS × Health + Envir	0.405	(0.309)	Neophilia × Enviro	−0.147	(0.208)
Income 2	0.328 **	(0.156)	Neophobia × Enviro	0.307 *	(0.185)
Income 3	0.746 ***	(0.245)	Neophilia × Health + Envir	−0.250	(0.185)
Income 4	1.041 ***	(0.223)	Neophobia × Health + Envir	0.076	(0.177)
College	0.899 ***	(0.329)	Income 2	0.327 **	(0.155)
Graduate	0.620	(0.408)	Income 3	0.754 ***	(0.246)
Age 2	0.001	(0.150)	Income 4	1.041 ***	(0.227)
Age 3	0.933	(0.699)	College	0.861 ***	(0.328)
Gender	−0.089	(0.145)	Graduate	0.583	(0.407)
			Age 2	−0.004	(0.150)
			Age 3	0.892	(0.694)
			Gender	0.327	(0.144)
Log Likelihood	−4118.942		Log Likelihood	−4114.023	
Wald (*p*-value)	218.89 (0.00)		Wald (*p*-value)	224.66 (0.00)	
N	1536		N	1536	

***, ** and * indicate significance at the 1%, 5% and 10% levels, respectively; The values in parentheses are robust standard errors.

### 5.3. Robustness Test

To address potential estimation bias from omitted variables and validate the robustness of the baseline results, two robustness checks were conducted: (i) by changing the econometric model and (ii) by using alternative subsamples (Table 9 and Table 10). The first two columns of these tables, respectively, display the robustness test results after aggregating the scores of the FNS scale and after splitting it into two factors. This study first conducted robustness tests by altering the econometric model. The Tobit models with left-censoring at zero were employed as an alternative specification [44]. The results in Table 9 indicate that the Log Likelihood values for the two models are −3327.603 and −3327.500, respectively, while the Wald χ^2^ (*p*-value) statistics are 25.97 (0.00) and 23.56 (0.00), respectively. These results indicate that both models exhibit satisfactory fitting performance. Additionally, the direction and significance level of the coefficient for FNS did not change substantially. Neophilia continued to positively influence WTP, while neophobia retained a significant negative effect. These results further confirm the robustness of the findings.

**Table 9:** Results of robustness test for the replacement model.

	(1)		(2)		
	Coef.	Robust Std.Err.		Coef.	Robust Std.Err.
FNS	−1.233 ***	(0.091)	Neophilia	0.589 ***	(0.077)
			Neophobia	−0.642 ***	(0.072)
Income 2	0.250 **	(0.125)	Income 2	0.253 **	(0.125)
Income 3	0.618 ***	(0.204)	Income 3	0.621 ***	(0.204)
Income 4	0.830 ***	(0.186)	Income 4	0.823 ***	(0.188)
College	0.778 ***	(0.248)	College	0.778 ***	(0.248)
Graduate	0.560 *	(0.314)	Graduate	0.562 *	(0.314)
Age 2	0.038	(0.120)	Age 2	0.041	(0.121)
Age 3	0.984 *	(0.587)	Age 3	0.973 *	(0.586)
Gender	−0.052	(0.115)	Gender	−0.051	(0.115)
Log Likelihood	−3327.603		Log Likelihood	−3327.500	
Wald (*p*-value)	25.96 (0.00)		Wald (*p*-value)	23.56 (0.00)	
N	1536		N	1536	

***, ** and * indicate significance at the 1%, 5% and 10% levels, respectively; The values in parentheses are robust standard errors.

We further conducted robustness checks using subsample regression, excluding respondents aged 50 and above (n = 74), in line with hypothesis that older individuals may exhibit stronger food neophobia and potential cognitive bias [43]. The results are presented in Table 10. The first and second columns of the table, respectively, display the robustness test results for the FNS scale after score summation and its division into two factors. The Log Likelihood values for the two models are −3916.160 and −3915.826, respectively, and the Wald χ^2^ (*p*-value) statistics are 186.47 (0.00) and 188.97 (0.00), respectively. These results indicate that both models exhibit strong explanatory power and a good overall fit. The signs and significance of the core explanatory and demographic variables remained stable, further confirming the robustness of the findings.

**Table 10:** Results of robustness test for replacement samples.

(1)			(2)		
	Coef.	Robust Std.Err.		Coef.	Robust Std.Err.
FNS	−1.521 ***	(0.123)	Neophilia	0.699 ***	(0.099)
			Neophobia	−0.819 ***	(0.092)
Income 2	0.420 ***	(0.157)	Income 2	0.426 ***	(0.157)
Income 3	0.767 ***	(0.239)	Income 3	0.775 ***	(0.238)
Income 4	1.069 ***	(0.225)	Income 4	1.052 ***	(0.229)
College	1.259 ***	(0.365)	College	1.263 ***	(0.364)
Graduate	0.975 **	(0.436)	Graduate	0.983 **	(0.435)
Age	0.006	(0.011)	Age	0.006	(0.011)
Gender	−0.115	(0.148)	Gender	−0.113	(0.148)
Log Likelihood	−3916.160		Log Likelihood	−3915.826	
Wald (*p*-value)	186.47 (0.00)		Wald (*p*-value)	188.97 (0.00)	
N	1462		N	1462	

*** and ** indicate significance at the 1% and 5% levels, respectively; The values in parentheses are robust standard errors.

## 6. Conclusions and Implications

### 6.1. Conclusions

Drawing on online survey data of Chinese consumers, this study employed the payment card method to estimate consumers’ WTP for the hybrid food beef rice. From a behavioral economics perspective, it further investigated the mechanisms through which food neophobia influences payment behavior. Using factor analysis, neophobia was divided into two dimensions: food neophobia and food neophilia. A multidimensional regression model was constructed accordingly. This research deepens the theoretical understanding of consumer psychology in the context of hybrid foods and offers empirical insights to guide public policy and marketing strategies.

First, our research findings indicate that Chinese consumers exhibit a generally positive attitude toward hybrid foods such as beef rice, with higher WTP compared to traditional rice. The estimated average WTP reflects a 16.4% premium over the prevailing market price of traditional rice. This suggests that beef rice serves as a cost-effective and nutritionally advantageous option for Chinese consumers undergoing dietary restructuring and seeking higher protein intake. These findings indicate that hybrid foods have the potential for market success, particularly amid rising interest in nutritional enhancement and food innovation among Chinese consumers.

Second, Chinese consumers exhibit food neophobia, which significantly suppresses their WTP for beef rice, but the effect varies across dimensions. The regression results indicate a significant negative correlation between consumers’ food neophobia and their WTP, suggesting that an increase in food neophobia is associated with a decrease in WTP. However, the results of the dimension-specific regression analysis based on factor analysis reveal that the effect of food neophobia on WTP exhibits significant heterogeneity across different dimensions. Specifically, Chinese consumers’ food neophilia significantly enhances their WTP, whereas their food neophobia significantly reduces it.

Third, environmental information significantly moderates the negative effect of food neophobia on WTP, whereas health information and combined framings do not exhibit significant additional effects. Environmental messages may enhance WTP by reducing perceived risks and highlighting sustainability advantages, such as lower carbon emissions and water use. However, the combined information treatment did not show a significant moderating effect. This may be due to cognitive overload or conflicting motivations caused by presenting multiple messages simultaneously, although this interpretation remains tentative. Moreover, the moderating effects differed by neophobia dimension: while no significant effects were observed in the food neophilia dimension, single-information framings (health or environment) exhibited significant positive effects on the food neophobia dimension, whereas combined framings did not.

### 6.2. Implications

Based on the above findings, two key recommendations are proposed for promoting the acceptance and commercialization of hybrid foods such as beef rice. First, tailored consumer education and targeted communication strategies should be developed for individuals with high food neophobia toward hybrid foods like beef rice. Given that neophobia significantly reduces WTP, evidence-based risk communication is essential to improve consumer trust. We recommend disseminating scientifically grounded information about beef rice and similar hybrid foods through television, social media platforms, and other mainstream channels. Since beef rice offers combined nutritional, environmental, and economic benefits, particular attention should be directed toward low-income and nutritionally vulnerable populations. Second, information interventions should be implemented via channel-specific and message-isolated strategies. This study found that single-information framings (health or environmental) are more effective than combined framings. Therefore, we recommend adopting a message separation strategy: health-related information can be emphasized in pharmacies and community health centers, while environmental benefits may be highlighted in organic markets, environmental exhibitions, or eco-label campaigns.

### 6.3. Limitations

This study also has certain limitations. The proportion of respondents with a bachelor’s degree in our sample is as high as 86.7%, whereas individuals with a high school diploma or postgraduate qualifications constitute a very small portion. This sampling bias may limit the generalizability of the findings to the broader consumer population. Nevertheless, we made every effort to ensure the randomness and validity of the data throughout the research process, and similar sampling patterns have also been documented in prior research [45,46]. Moreover, as our analysis is based on survey data, it may be subject to certain biases [47]. Nevertheless, the stated purchasing behavior in hypothetical scenarios can uncover trends that are not readily observable in market data, offering meaningful implications for the future commercialization of beef rice.

## Figures and Tables

**Table 1:** Description of information treatments provided to experimental groups.

Treatments	Information Content
Benchmark	Message 1. Introduction to beef rice. Beef rice is a novel food product developed in the laboratory. Researchers apply an edible coating composed of fish gelatin and food-grade enzymes to the surface of rice grains, allowing beef cells to attach and grow. It is distinct from traditional beef mixed with rice and does not involve conventional cattle farming or slaughter. Importantly, beef rice is not a genetically modified food. The current standard indicates that each jin (500 g) of beef rice contains approximately 2.4 g of beef cells. After cooking, it has a slightly firmer texture than traditional rice, with a mild meaty and nutty aroma. Beef rice has not yet been introduced into the Chinese market.
Health information	Message 1. Includes all content from the benchmark group. Message 2. Health-related information. Compared with traditional rice, beef rice contains approximately 8.66% more protein and 7.14% more fat, while other nutrients such as carbohydrates are similar.
Environment information	Message 1. Includes all content from the benchmark group. Message 3. Environmental information. To produce 100 g of protein, traditional beef generates approximately 49.89 kg of CO_2_-equivalent emissions, rice generates around 6.27 kg, and beef rice emits even less than rice, while also saving land and freshwater resources.
Health and environment information	Message 1. Includes all content from the benchmark group. Message 2. Both health and environmental framing as described above. Health framing: beef rice offers higher protein (+8.66%) and slightly higher fat (+7.14%) than traditional rice. Message 3. Environment: CO_2_ emissions per 100 g of protein are significantly lower than those of traditional beef (49.89 kg) and even lower than rice (6.27 kg), offering benefits in both carbon reduction and resource conservation.

**Table 2:** Definitions and coding of variables.

Variable	Variable Name	Variable Definition and Coding
Dependent variable	WTP	The price range consumers are willing to pay per jin of beef rice
Independent variable	FNS (overall dimension)	Consumers’ scores on the FNS
	Food neophilia (sub-dimension 1)	Factor analysis scores of items 1, 4, 6, 9, and 10
	Food neophobia (sub-dimension 2)	Factor analysis scores of items 2, 3, 5, 7, and 8
Control variable	Gender	Male = 1; Female = 0
	Age	Age 1: 30 years old and under = 1; others = 0
		Age 2: 30 to 60 years old = 1; others = 0
		Age 3: Over 60 years old = 1; others = 0
	Education	High school: High school and below = 1; others = 0
		College: University and junior college = 1; others = 0
		Graduate: Postgraduate and above = 1; others = 0
	Annual income (RMB)	Income 1: ≤200,000 yuan = 1; others = 0
		Income 2: 200,000 to 400,000 yuan = 1; others = 0
		Income 3: 400,000 to 600,000 CNY = 1; others = 0
		Income 4: >600,000 CNY = 1; others = 0
	Information treatments	Health: Health information processing = 1; others = 0
		Envir: Environmental information processing = 1; others = 0
		Health + Envir: Health and environmental information processing = 1; others = 0

**Table 3:** Demographic characteristics of the sample (n = 1536).

Variable	Sample Structure		
	Parameter	Frequency	Percentage (%)
Gender	Male	940	61.198
	Female	596	38.802
Annual pre-tax household income	Under 200,000 CNY	525	34.180
	200,000–400,000 CNY	660	42.969
	400,000–600,000 CNY	220	14.323
	Over 600,000 CNY	131	8.529
Education	High school or below	73	4.753
	University or junior college	1331	86.654
	Postgraduate or above	132	8.594
Age	30 years old or younger	452	29.427
	30–60 years old	1069	69.596
	Over 60 years old	15	0.977

**Table 4:** Exploratory factor analysis of FNS items: means, loadings, and explained variance.

Items		Mean	Std.Dev.	Neophilia	Neophobia
1R ^a^	I will constantly try different new foods.	2.329	0.972	**0.816R ^b^**	0.172
2	I don’t trust new foods.	2.446	0.954	−0.282	**0.722**
3	If I don’t know what a food is, I won’t try it.	3.233	1.113	0.015	**0.750**
4R	I like foods from different countries.	2.400	0.999	**0.760**	−0.015
5	Foods with strange appearances won’t be eaten by me.	2.837	1.088	−0.141	**0.767**
6R	At banquets, I will try new foods.	2.135	0.915	**0.824**	−0.086
7	I’m afraid to eat things I’ve never eaten before.	2.519	1.030	−0.217	**0.750**
8	I’m very picky about the food I’m about to eat.	2.878	0.973	0.106	**0.662**
9R	I eat almost everything.	2.934	1.131	**0.544**	−0.117
10R	I like to try new niche restaurants.	2.294	0.929	**0.759**	−0.016

a: R Denote reverse scoring; b: Higher loadings on any factor are shown in bold.

**Table 5:** Reliability test results for the FNS scores.

Dimension	Items	CITC	Cronbach’s α If Item Deleted	Cronbach’s α	Cumulative Variance Contribution Rate (%)
Food neophilia	FNS1	0.682	0.729	0.799	29.533%
	FNS4	0.582	0.760		
	FNS6	0.679	0.733		
	FNS9	0.420	0.819		
	FNS10	0.587	0.760		
Food neophobia	FNS2	0.603	0.742	0.791	56.768%
	FNS3	0.561	0.756		
	FNS5	0.619	0.735		
	FNS7	0.452	0.787		
	FNS8	0.621	0.735		

**Table 6:** Confirmatory factor analysis results: factor loadings, CR, and AVE.

Path Relationship		Factor Loading	S.E.	AVE	CR
FNS1	Factor 1: Food Neophilia	0.802		0.472	0.818
FNS4		0.665	0.034		
FNS6		0.791	0.031		
FNS9		0.464	0.039		
FNS10		0.662	0.032		
FNS2	Factor 2: Food Neophobia	0.734		0.438	0.796
FNS3		0.605	0.046		
FNS5		0.713	0.046		
FNS7		0.739	0.044		
FNS8		0.481	0.040		

## Data Availability

The data presented in this study are available on request from the corresponding author. The data are not publicly available due to being a part of an ongoing study.
